# Potential Clinical Implications of miR-1 and miR-21 in Heart Disease and Cardioprotection †

**DOI:** 10.3390/ijms21030700

**Published:** 2020-01-21

**Authors:** Branislav Kura, Barbora Kalocayova, Yvan Devaux, Monika Bartekova

**Affiliations:** 1 Institute for Heart Research, Centre of Experimental Medicine, Slovak Academy of Sciences, 84104 Bratislava, Slovakia; branislav.kura@savba.sk (B.K.); barbora.kalocayova@savba.sk (B.K.); 2 Institute of Physiology, Faculty of Medicine, Comenius University in Bratislava, 81372 Bratislava, Slovakia; 3 Cardiovascular Research Unit, Department of Population Health, Luxembourg Institute of Health, L-1445 Strassen, Luxembourg; yvan.devaux@lih.lu

**Keywords:** microRNA-1 (miR-1), microRNA-21 (miR-21), cardiovascular diseases, cardioprotection, biomarkers

## Abstract

The interest in non-coding RNAs, which started more than a decade ago, has still not weakened. A wealth of experimental and clinical studies has suggested the potential of non-coding RNAs, especially the short-sized microRNAs (miRs), to be used as the new generation of therapeutic targets and biomarkers of cardiovascular disease, an ever-growing public health issue in the modern world. Among the hundreds of miRs characterized so far, microRNA-1 (miR-1) and microRNA-21 (miR-21) have received some attention and have been associated with cardiac injury and cardioprotection. In this review article, we summarize the current knowledge of the function of these two miRs in the heart, their association with cardiac injury, and their potential cardioprotective roles and biomarker value. While this field has already been extensively studied, much remains to be done before research findings can be translated into clinical application for patient’s benefit.

## 1. Introduction

Despite significant progress in cardiovascular research and improvements in the management of patients, cardiovascular disease (CVD) is still the leading cause of death worldwide. In addition to major diseases caused by ischemic injury including ischemic heart disease and myocardial infarction, non-ischemic cardiac disorders such as inflammatory cardiomyopathy, anthracycline-induced cardiomyopathy, diabetic cardiomyopathy, or radiation-induced heart disease largely contribute to overall disability and mortality due to CVD. Thus, searching for new therapeutic targets that may lead to new treatments for CVD based on deep molecular research, including transcriptomic research exploring the role of different RNAs in CVD, is very much needed.

In addition to numerous other mechanisms and molecules, there is emerging evidence that non-coding RNAs (ncRNAs) play an important role in heart physiology and function including cardiac development, aging, heart disease, and cardioprotection [1,2,3,4,5]. NcRNAs represent a large family of RNA molecules that, in contrast to messenger RNAs (mRNAs), are not directly involved in gene transcription to proteins but widely contribute to regulation of gene expression and protein synthesis, thus regulating numerous processes in the cell. The most important and studied subgroup of ncRNAs are microRNAs (miRNAs, miRs), a class of small ncRNAs of ∼22 nucleotides in length. MiRs are involved in the regulation of gene expression at the post-transcriptional level by degrading their target mRNAs and/or by inhibiting the translation, thus controlling many different processes and pathways within the cell [6]. Hundreds of different miRs have been identified so far, and their functions in different organs and systems including the cardiovascular system are widely studied both under physiological and pathological conditions. Various miRs have been documented to be involved in the development of different types of CVD including myocardial infarction [7], drug-induced cardiotoxicity [8], as well as different cardiomyopathies [9,10]. In line with their role in CVD pathogenesis, and reflecting the fact that numerous miRs are released into the circulation in CVD, miRs are potential biomarkers of CVD. However, translation of experimental knowledge to clinical application is complicated and the routine use of circulating levels of miRs in the clinic is still far away.

Due to the high number of experimental studies revealing the role of different miRs in health and disease including their role in CVD, there is a need to compare the studies and summarize the knowledge about the role of specific miRs in the development of CVD as well as in cardiac protection and repair. This will allow drawing an overall picture of the pathophysiological function of miRs. Hence, this review article summarizes the current knowledge on the role of two miRs which have been documented to play significant roles in CVD, miR-1, and miR-21. We will review their role in the pathogenesis and progression of different types of CVD, as well as in cardiac protection and repair. Finally, we will discuss their potential as CVD biomarkers.

## 2. MiR-1 and miR-21: Discovery, Structure, Biology, and Physiological functions

MiRs are present and encoded in the genomes of all plants and animals, as well as in some viruses. The mechanism of their action consists of binding to the 3’ untranslated region (3’UTR) of the specific messenger, RNA (mRNA), at the post-transcriptional level [11]. MiRs are small (∼19–25 nucleotides), evolutionary conserved, non-coding, single-stranded RNA molecules. After binding of miRs to specific mRNA, the cleavage of the mRNA strand into two parts, the loss of mRNA stability, and hampered translation of the mRNA into proteins occur [12,13,14,15]. It is supposed that miRs can modulate at least 30% of the human protein-coding genome [16]. One of the characteristics of miR is that each miR can influence several targets and more than one miR can affect a single mRNA. According to many studies and experiments in different animal models, it is assumed that miRs play a critical role in the regulation of many biological processes including cell differentiation and proliferation, apoptosis, as well as cell cycle progression [12,16,17,18].

MiRs were discovered in 1993 by Ambros and colleagues. The first miR, lin-4, was isolated from nematode *Caenorhabditis elegans* [19]. Up to now, almost 2000 miRs have been identified and described in humans (http://www.miRbase.org—27.11.2019) and more than 200 miRs have been found in the heart or associated with cardiovascular diseases [20].

**Figure 1:**
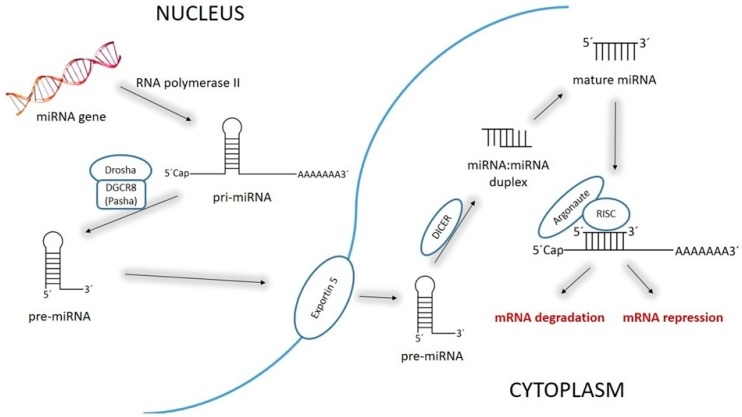
Process of microRNAs (miRs) synthesis, processing, and targeted miR–binding. MiR synthesis occurs in the nucleus and the cytoplasm. In the nucleus, the primary miR transcript (pri-miR) is processed to pre-miR. In the cytoplasm, pre-miR is transformed to the mature miR.

Synthesis of miR starts with the transcription of miR genes by RNA polymerase II. This process of transcription leads to the formation of primary miR transcript (pri-miR) [21]. By the action of microprocessor complex, which contains two important parts—the double-stranded RNase III enzyme, DROSHA, and cofactor, the DiGeorge syndrome critical region 8 (DGCR8)—production of a hairpin structure precursor miR (pre-miR) in the nucleus occurs [15]. These pre-miRs are double-stranded, circa 70 nucleotides long, and they have a terminal loop. After this, pre-miRs are relocated by exportin-5 to the cytoplasm for additional processing. In the cytoplasm, the RNase III enzyme, DICER, cleaves a hairpin, resulting in the formation of a mature, circa 22 nucleotides long miR:miR duplex [22]. Helicase transforms miR duplexes into single-stranded miRs. Together with the Argonaute (AGO) protein, they create the multiprotein RNA-induced silencing complex (RISC) inside which one strand represents the mature miR and the other is degraded. Within this complex, miRs inhibit the translation of specific mRNAs or give rise to their destabilization and degradation [11,18,21,22]. The process of miRs synthesis, maturation, and targeting miR–binding is summarized in Figure 1.

MiR-1 is considered as a muscle-specific miR. There are two different precursors of miR-1 in humans, miR-1-1 and miR-1-2, both of which are processed into an identical mature form of miR-1 [23]. Usually, it exists in a cluster with miR-133. It was observed that it is preferentially expressed in cardiac and skeletal muscles and, according to Rao et al. [24], miR-1 is the most abundant miR in the adult mouse heart since embryonal development. Early experiments with modulation of miR-1 expression resulted in significant embryonic defects leading to many cardiovascular disorders, or to the death of animals. MiR-1, together with a few other miRs, have a significant role in the development of embryonic stem cells and cardiomyocyte progenitor cells [25]. It is also connected with the expression of many cardiac transcription factors like myocardin, Nkx2.5, serum response factor (SRF), WNT, and FGF signaling pathways. Cyclin-dependent kinase-9 (Cdk9), histone deacetylase 4 (HDAC4), Sox6, FZD7 (Frizzled-7), and FRS2 (fibroblast growth factor receptor substrate 2) are considered targets of miR-1 genes that are involved in cardiac development or function [26,27].

In recent years, miR-21 has received increased attention due to its involvement in many biological processes, mainly in CVD. Deregulated expression of miR-21 was observed also in the heart and vasculature under CVD conditions such as proliferative vascular disease, cardiac hypertrophy and heart failure, and ischemic heart disease [28]. MiR-21 belongs to the miRs that are most abundantly expressed in the organism. Changed expression of miR-21 was observed in different kinds of diseases and damaged tissues like CVD, pulmonary diseases, inflammation-induced diseases, or oncological diseases, and it is also included in immunological and developmental processes [29]. It was proved that miR-21 acts as an anti-apoptotic and pro-survival factor in different cells [30,31]. Up-regulated expression of miR-21 was observed in cancer cells and it is considered as a common attribute of pathological cell growth and cell stress [32]. MiR-21 exhibited higher expression levels in mice with cardiac hypertrophy [33]. Pan et al. [34] identified several conserved enhancer elements in the sequence of miR-1, including binding sites for activation protein 1 (AP-1), Ets family transcription factor PU.1, CCAAT/enhancer-binding protein-α, nuclear factor I (NFI), serum response element, p53 and signal transducer, and activator of transcription 3 (STAT3). Further potential targets of miR-21 were detected like AP-1 and programmed cell death protein 4 (PDCD4) in solid and hematological malignancies [35].

## 3. Role of miR-1 and miR-21 in Ischemic Injury of the Heart

Ischemia-reperfusion (I/R) injury of the heart is a major CVD associated with such clinical manifestations and situations as ischemic heart disease and acute myocardial infarction (AMI), as well as with heart surgery and transplantation. Ischemia is caused by obstruction in the coronary artery leading to the reduction or stopping of blood flow to the heart tissue and finally leading to insufficient oxygen and metabolic supply. Reperfusion is the restoration of the blood flow, which in case that is maintained too late, may even exacerbate ischemic injury. Numerous mechanisms have been documented to be involved in the development and progression of cardiac I/R injury including different intracellular, extracellular, as well as systemic changes in response to ischemic insult. These changes lead to altered homeostasis within the heart as well as to systemic changes due to the release of different molecules to circulation. The final state of the heart depends on the intensity and duration of ischemic stimuli as well as on the presence/absence of protective interventions to ischemic heart. Major mechanisms have been suggested to play a crucial role in the development of I/R injury are oxidative stress and inflammation [36,37]; however, other mechanisms such as activation of matrix metalloproteinases, activation of apoptosis, alterations in gene expression, etc., have been documented to be involved as well [38,39,40]. In the last decade, there has been emerging evidence documenting changes in various types of non-coding RNAs (ncRNAs) including miRs [41,42], long non-coding RNAs (lncRNAs) [2,43,44], and circular RNAs (circRNAs) [45,46,47] due to myocardial infarction, thus suggesting that these molecules may be involved in the mechanism of cardiac I/R injury and may potentially be candidates for therapeutic targets and/or biomarkers of cardiac I/R injury, including AMI.

In addition to other ncRNA types, numerous miRs have been documented to be associated with the development of cardiac I/R injury as well as with post-ischemic remodeling of the heart. Among them, miR-1 and miR-21 have been extensively studied and well documented, although their exact role in I/R injury is still not fully clarified. The aim of this part of the paper is to summarize the current knowledge on the involvement of these two particular miRs in the development and progression of cardiac I/R injury based on recent findings from in vitro studies in cell cultures of cardiac myocytes, as well as in ex vivo and in vivo studies in AMI animal models and human studies in AMI patients and patients undergoing ischemia-associated cardiac surgery.

### 3.1. Role of miR-1 in Cardiac I/R Injury

MiR-1 is a muscle-specific miR reported to be tightly associated with CVD. Regarding I/R injury, miR-1 was found to be down-regulated in response to myocardial I/R injury in the heart tissue of rats [48,49] and mice [50], as well as in cardiac-derived H9c2 cells [48] and neonatal cardiac myocytes [51] exposed to hypoxia/reoxygenation (H/R). The level of miR-1 down-regulation was increased with prolonged time of MI in rats, and also with prolonged time of post-hypoxic reoxygenation in H9c2 cells [48]. MiR-1 was found to be down-regulated also in heart tissues obtained by post-mortem autopsies from infarcted human hearts [52,53]. When the infarcted hearts were divided into subgroups according to the duration of MI, tissue expression of miR-1 was down-regulated only in the subgroup with more than one day and less than seven days old MI, in which signs of total coagulation necrosis and loss of nuclei accompanied by edema and neutrophil infiltration of the interstitium was observed [52]. The findings suggest that there is a time-dependent alteration in miR-1 expression during the process of I/R injury and/or post-infarction tissue remodeling due to MI. In contrast, it was documented that miR-1 is up-regulated in remote myocardium when compared to infarcted tissue or healthy adult hearts [54]. The important role of miR-1 in I/R injury was proven in an animal model of I/R injury using transgenic mice overexpressing miR-1 and treatment with locked nucleic acid-modified oligonucleotide against miR-1 (LNA-anti-miR-1) to suppress miR-1 expression. It was found that while miR-1 overexpression exacerbated myocardial I/R injury manifested by increased infarct size, apoptosis and caspase-3 expression in the hearts, as well as elevated serum LDH and CK levels, LNA-anti-miR-1 treatment attenuated I/R injury [55]. In concordance, miR-1 inhibition has been shown to protect rat H9c2 cardiomyocytes against H/R-induced apoptosis likely via targeting Bcl-2 [48]. Inhibition of miR-1 was found protective against cardiac I/R injury also in rats likely via promoting MAPK3/PI3K/Akt signaling [56]. In a comparative study in young and old mice exposed to MI, increased levels of miR-1 were found in old mice post-MI in comparison to young ones, and this was associated with enhanced adverse cardiac remodeling, suggesting elevated levels of miR-1 as a predictive biomarker of high-risk of myocardial injury in older individuals [57]. Since elevated miR-1 predicts an increased risk of post-ischemic injury, it seems that the down-regulation of tissue miR-1 due to I/R or MI might represent an adaptive mechanism of the heart to the ischemic conditions. In contrast to widely documented reduced levels of miR-1 in the heart tissue, levels of circulating miR-1 were significantly increased in AMI patients [58], in ST segment elevation myocardial infarction (STEMI) patients undergoing primary percutaneous coronary intervention, as well as in a pig model of AMI [59]. Interestingly, circulating miR-1 levels strongly correlated with the rate of glomerular filtration indicating renal elimination in STEMI patients, which was finally confirmed by the detection of miR-1 in urine [59]. The increase in serum and urine levels of miR-1 were found also in patients undergoing open-chest surgeries with cardiopulmonary bypass (CPB) when these levels were increased both 60 min and 24 h post-CBP. A similar increase in serum troponin-I and a strongly positive correlation with miR-1 was identified in these patients, suggesting miR-1 to be a potentially novel biomarker for myocardial injury in heart surgery [60]. On the other hand, in a recent study in STEMI patients and patients with cardiogenic shock (CS), circulating levels of miR-1 were elevated only in CS patients while no changes in circulating miR-1 were observed in STEMI patients, thus making the potential of miR-1 as biomarker of acute myocardial injury less optimistic [61].

**Figure 2:**
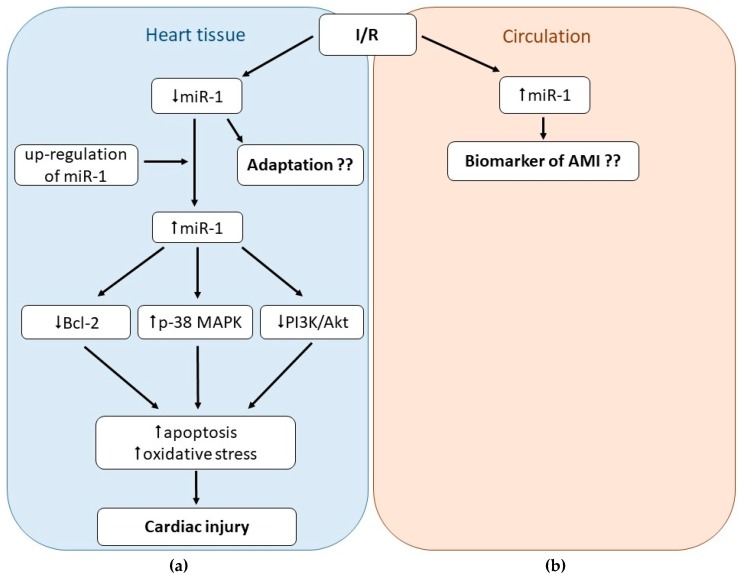
Role of microRNA-1 (miR-1) in cardiac ischemia-reperfusion (I/R) injury. MiR-1 seems to play diverse roles in I/R injury. In the heart tissue, miR-1 is down-regulated due to I/R (**a**) but up-regulated in circulation (**b**), thus suggesting miR-1 as a potential biomarker of I/R injury. Up-regulation of miR-1 in the heart tissue (e.g., in transgenic animals or older patients) seems to exacerbate I/R injury via promoting oxidative stress and apoptosis (**a**).

Taken together, miR-1 is intimately associated with myocardial I/R injury; however, its exact role still remains not fully clarified. On one side, I/R injury is associated with the down-regulation of miR-1 in the heart tissue or isolated cardiac cells; on the other hand, increased tissue levels of miR-1 are associated with increased cardiac damage due to I/R. It also seems that tissue levels of miR-1 may vary depending on the time duration of MI and/or post-ischemic reperfusion and cardiac remodeling post-MI. The impact of miR-1 expression on arrhythmogenesis post-MI is also controversial; elevated miR-1 was found both pro- and anti-arrhythmogenic. Finally, circulating levels of miR-1 were proven to be elevated due to cardiac I/R thus being proposed to be potentially served as biomarkers of cardiac I/R injury; however, the cardiac and disease specificity of this RNA should be carefully considered. In the overall picture (Figure 2), it seems that in ischemic conditions or post-MI, miR-1 is released from the heart tissue to circulation, which may represent an adaptive mechanism of the heart to ischemia, since elevated levels of miR-1 exacerbate myocardial damage due I/R.

### 3.2. Role of miR-21 in Cardiac I/R Injury

MiR-21 also belongs to miRs associated with different kinds of CVD including myocardial I/R injury [28]. It was reported that tissue expression of this miR is changed in the early phase of AMI (six hours after AMI) in rats, and these changes might depend on the zone of the infarcted heart. While miR-21 expression was down-regulated in infarcted areas, it was up-regulated in borderline areas [62]. I/R-induced up-regulation of miR-21 was found also on days two and seven post-MI in mice, but in this study, miR-21 was specifically localized in the infarct region of the heart [63]. Further, it was documented that miR-21 levels significantly decreased in the first week and second week post-MI, but were almost unchanged in the fourth week in a mouse model of AMI [64]. These data suggest that changes in tissue expression of miR-21 due to MI may depend on the time of MI and localization within the heart. MiR-21 was found up-regulated also in different cardiac cells exposed to hypoxia, including mouse cardiomyocytes, H9c2 and HL-1 cells, and human HCM cardiomyocytes [65]. In the same study, inhibition of miR-21 increased cell apoptosis associated with reduced hypoxia-inducible factor 1-alpha (HIF-1α) expression, suggesting that there is a tight connection between HIF-1α and miR-21 in myocardial I/R injury [65]. A recent study showed that miR-21 was down-regulated in rat neonatal cardiomyocytes exposed to simulated ischemia (oxygen-glucose deprivation). This was associated with the up-regulation of its target gene, *PDCD4*, an increased number of apoptotic cells, and reactive oxygen species (ROS) [66]. While inhibition of miR-21 expression in MI seems to have detrimental effects on the heart, overexpression of this miR was found to be cardioprotective. Adenovirus-mediated gene transfer-induced overexpression of miR-21 decreased infarct size 24 h after AMI, and this cardioprotective effect of miR-21 was likely mediated by targeting the PDCD4 gene and its downstream molecule, AP-1 [62]. Adenovirus miR-21 also improved LV remodeling and decreased the apoptosis of myocardial cells in a rat model of myocardial I/R injury [67]. Transfection of miR-21 expressing lentivirus into the left ventricular cavity of mice induced cardioprotection against I/R injury manifested by decreased infarct size, fibrosis, and apoptosis [64]. In human cardiac myocytes transfected with the miR-21 mimic short hairpin RNA (shRNA), the apoptosis rates were down-regulated compared with control cells, and this was associated with down-regulated protein expression of p-JNK, p-p38 MAPK, and caspase-3, suggesting that miR-21 inhibits tumor necrosis factor-alpha (TNF-α)-induced apoptosis by activating the JNK/p38-MAPK/caspase-3 signaling pathway [68]. The cardioprotective effect of miR-21 was proved also in the recent study by Chen et al. [69] where depletion of exosomal miR-21 from cardiomyocyte-derived conditioned medium (CM) reduced the protective effect of CM on cardiomyocytes against H_2_O_2_-induced oxidative stress, enhanced fibroblast activation, and reduced angiogenesis in endothelial cells. Depletion of miR-21 also reduced the effect of CM on rat hearts exposed to AMI, manifested by attenuation of CM-induced reduction of infarct size and immune cell infiltration [69]. The role of exosomal miR-21 was documented also in the study by Xiao et al. [70] where miR-21 was significantly up-regulated in oxidative stress-induced cardiac progenitor cell (CPC)-derived exosomes, and these exosomes prevented cardiomyocytes apoptosis in H9c2 cells, likely via inhibition of PDCD4.

Post-MI cardiac remodeling is associated with the development of fibrosis in the heart tissue, and miR-21 has been documented to be involved in this process. Yuan et al. [71] documented that miR-21 is elevated in the infarct zone in mouse hearts exposed to AMI and promotes myocardial fibrosis post-MI. In the same study, transforming growth factor-beta 1 (TGF-β1) treatment enhanced miR-21 expression in cardiac fibroblasts, and overexpression of miR-21 promoted the TGF-β1-induced fibroblast activation evidenced by increased expression of Collagen-1, alpha-smooth muscle actin (α-SMA), and F-actin, whereas inhibition of miR-21 attenuated fibrotic process. Finally, they detected Smad7 as a direct target of miR-21, altogether suggesting that miR-21 may play a key role in cardiac fibrosis post-MI via TGF-β/Smad7 signaling [71]. The important role of miR-21 in the pathogenesis of cardiac fibrosis has been shown also in heart atria from patients with atrial fibrillation likely via targeting its downstream target Sprouty 1 (Spry1) [72]. In the study, miR-21 expression in heart atria was significantly increased in these patients compared to patients with sinus rhythm, and this increase positively correlated with atrial collagen content and was associated with reduced protein expression of Spry1. Further, a decrease of miR-21 by antagomir-21 prevented fibrosis of the atrial myocardium post-myocardial infarction, altogether indicating the critical role of miR-21 in atrial fibrosis post-MI [72]. Very similar results confirming the pro-fibrotic role of miR-21 in atrial fibrillation via targeting Spry1 have been obtained in a rat experimental model of AMI [73]. Another mechanism involved in the miR-21 mediated development of cardiac fibrosis is targeting Jagged1 [74]. Recently, nanoparticle delivery of miR-21 mimics to cardiac macrophages post-MI has been shown to promote angiogenesis and reduce hypertrophy, fibrosis, and cell apoptosis in the remote myocardium, suggesting a new therapeutic strategy using nanoparticle delivery of miR-21 to attenuate post-MI remodeling and heart failure [75].

In addition to altered tissue expression of miR-21 due to I/R or AMI, circulating levels of miR-21 were also found to be changed in MI. For example, diverse changes in circulating miR-21 levels have been found in 12 post-MI patients; miR-21 initially decreased two days post-MI, increased five days post-MI, and returned to values of age-matched referent controls later post-MI time points [76]. Circulating miR-21 levels were found elevated also in patients with coronary artery disease in response to cardiac stress, concretely at 24 h after dobutamine stress echocardiography [77]. Further, miR-21 expression in the serum of elderly patients (>65 years) with AMI was up-regulated compared to healthy controls, and levels of this miR were positively correlated with serum levels of CK-MB and cTnI [68]. On the other hand, unchanged concentrations of circulating miR-21 were found in patients undergoing transcoronary ablation of septal hypertrophy (TASH), a procedure mimicking AMI [78]. Importantly, detected levels of (not only) miR-21 may depend on the method of miR measurement. For instance, miR-1 levels statistically differed between STEMI patients and patients with stable coronary artery disease when quantified using novel chip-based digital PCR whereas classical qRT-PCR was unable to reach significance [79]. Another novel method, droplet digital PCR, detected no association between miR-21 levels and ischemia-reperfusion injury in STEMI patients [80], and also did not find miR-21 as a potentially relevant marker of coronary artery disease [81].

In conclusion, miR-21 seems to play an important regulatory role in the pathophysiology of MI despite the changes in its expression in MI may differ depending on the time and area within the heart tissue. In general, miR-21 exerts cardioprotective effects in MI likely via decreasing apoptosis induction. On the other hand, the role of miR-21 in post-MI cardiac remodeling seems controversial. While some studies found miR-21 to prevent fibrosis, the majority of studies suggested that miR-21 may contribute to the development of post-MI fibrosis in the infarcted heart tissue. Finally, data documenting the release of miR-21 into circulation post-MI are inconclusive, thus miR-21 would not be suggested as a potential biomarker of AMI. Findings documenting roles of miR-1 and miR-21 in I/R injury and other types of CVD are summarized in Table 1.

## 4. Role of miR-1 and miR-21 in Cardiac Arrhythmias

Cardiac arrhythmias belong to the most frequent heart pathologies that can happen at any age. They are characterized by irregular heart rhythm, either too fast (>100 beats/min) or too slow (<60 beats/min). There are different types of cardiac arrhythmias or abnormal heart beating, such as tachycardia, bradycardia, premature atrial or ventricular contractions, as well as the atrial or ventricular fibrillations. While most types of arrhythmia are not serious, some may lead to stroke or heart failure, or even result in sudden death. The pathogenesis of arrhythmias includes altered heart automaticity, triggered heart activity, or re-entry (electric signal does not complete the normal circuit). Various factors may influence heart automaticity and induce arrhythmias including ischemia, scarring, electrolyte disturbance, some medications, and old age [113]. Recently, it has been documented that several miRs including miR-1 and miR-21 may be involved in the pathogenesis of cardiac arrhythmias.

### 4.1. Role of miR-1 in Cardiac Arrhythmias

The potential role of miR-1 in arrhythmogenesis was firstly revealed by Yang et al. in 2007 [84], where authors documented that miR-1 is overexpressed in patients with ischemic heart disease and that overexpression of miR-1 in normal or infarcted rat hearts exacerbates arrhythmogenesis. Moreover, the down-regulation of miR-1 in infarcted rat hearts reduced arrhythmogenesis in the same study. Association between miR-1 up-regulation and the development of cardiac arrhythmias have since been later documented in other studies both in animals [85,86] and humans [87]. Mechanisms potentially involved in miR-1-mediated arrhythmogenesis include processes such as enhanced Ca^2+^ release [114], dissociation of phosphatase activity from RyR2 complex [85], altered expression of K^+^ channels [84,115], disturbed intracellular trafficking system [116], or altered expression of connexin-43 (Cx43) [84]. However, the exact mechanism by which miR-1 contributes to arrhythmogenesis is still to be elucidated.

The above-presented data indicate that the up-regulation of miR-1 contributes to arrhythmogenesis. Since miR-1 up-regulation has been shown also in patients with MI, it is suggested that miR-1 up-regulation might be responsible for the higher risk for arrhythmias in these patients [54]. On the other hand, the up-regulation of miR-1 expression by aldosterone blocker spironolactone was associated with reduced incidence of MI-associated ventricular arrhythmias in rats [117]. Furthermore, the heart tissue expression of miR-1was found reduced in patients with age-associated atrial fibrillation (AF) [88] and in patients with permanent AF undergoing heart surgery [89]. Thus, the role of miR-1 in cardiac arrhythmogenesis remains controversial.

### 4.2. Role of miR-21 in Cardiac Arrhythmias

Association of miR-21 and cardiac arrhythmias has been recently documented in several studies. Barana et al. [90] showed increased miR-21 expression in isolated human atrial myocytes from patients with chronic atrial fibrillation (CAF) as compared to myocytes from patients with sinus rhythm. Increased miR-21 expression in CAF was associated with decreased L-type calcium current in the study [90]. In contrast, lower plasma levels of miR-21 were found in patients with AF as compared to those without AF. Furthermore, plasma levels of miR-21 were lower in paroxysmal AF as compared to persistent AF. Atrial tissue expression of miR-21 was lower in patients with AF as compared to those without AF [91]. MiR-21 has been also shown to be associated with atrial fibrosis and remodeling during AF, where reduced miR-21 was associated with the prevention of atrial fibrosis and reduced prevalence of AF in mice [92]. Enhanced miR-21 expression was documented in a tachypaced as compared to non-paced normal rhythm hearts in a close-chest tachyarrhythmia model in pigs [118]. It was also shown that rapid atrial pacing induces myocardial fibrosis via increased miR-21 through down-regulating Smad7 in rabbit hearts [93]. MiR-21 was also shown to be involved in AF by promoting fibrosis in a sterile pericarditis model in rats, where a reciprocal loop between STAT3 and miR-21 was suggested to contribute to the development of AF [94]. A recent study by Tao et al. [119] documented that knockdown of miR-21 inhibits cardiac fibroblasts proliferation by inactivating the TGF-β1/Smad2 signaling pathway. Finally, it has been shown that circulating levels of miR-21 correlate with left atrial low-voltage areas and are associated with procedure outcome in patients with persistent AF undergoing ablation [74], and that the up-regulation of circulating miR-21 may indicate the presence of fibrosis in patients with left ventricular non-compaction cardiomyopathy (which symptoms may include arrhythmias) [120].

Taken together, altered miR-21 expression is associated with cardiac arrhythmias, mainly by playing a role in the atrial fibrosis development in AF, and circulating miR-21 might serve as a biomarker of fibrotic processes in arrhythmias-associated heart pathologies.

## 5. Role of miR-1 and miR-21 in Cardiomyopathies of Different Origin

Cardiomyopathy represents a group of diagnoses characterized by anatomic and functional changes of the heart associated with muscle and/or electrical dysfunction. They include heterogeneous cardiac disorders that often lead to advanced heart failure and significantly contribute to overall morbidity and mortality [121].

### 5.1. Role of miR-1 and miR-21 in Hypertrophic and Dilated Cardiomyopathy

Hypertrophic cardiomyopathy is defined as a myocardial disease characterized by hypertrophy of the septum and/or wall of the left ventricle and diastolic dysfunction of the left ventricle [10]. The complex roles of a single miR in different cell types in the heart have been uncovered. It was documented that miR-21 can promote cardiac fibrosis and cardiac hypertrophy in fibroblasts. On the other hand, miR-21 can protect cardiomyocytes against hypertrophy and apoptosis [97,122]. It was demonstrated that different cardiomyopathies had unique miR expression patterns. The expression of miR-21 and miR-1-3p showed disease specificity, which may provide new avenues for differentiating the hypertrophic and dilated cardiomyopathy. For example, the expression level of miR-1-3p had disease specificity and sensitivity in hypertrophic cardiomyopathy, whereas only miR-1-3p was associated with human left ventricular function in hypertrophic cardiomyopathy, suggesting it as a potential target for improvement of cardiac function in end-stage of this disease. The direct target of miR-1-3p is probably chloride voltage-gated channel 3 (Clcn3), which clarifies the mechanism of hypertrophic cardiomyopathy. The expression of miR-21 was up-regulated in dilated cardiomyopathy compared with control human hearts and no difference was found between hypertrophic cardiomyopathy and the control [10].

The latest research suggests that miR-21 could be the therapeutic target in non-ischemic cardiomyopathy. Mice hearts with transverse aortic constriction were accompanied by the increase of miR-21 levels while PDCD4 was lower. In neonatal rats, cardiomyocytes were AP-1-activated by angiotensin 2 (Ang2) stimulation, which conduced to an increase of miR-21 expression. In this study, the use of a miR-21-specific inhibitor was accompanied by increased levels of PDCD4, decreased AP-1 activity, and decreased expression of collagen type I and α-smooth muscle actin. These results indicate the important role of miR-21/PDCD4/AP-1 in the process of left ventricular remodeling in non-ischemic conditions [95]. It is interesting to note that miR-21 is differently expressed in the cardiomyocytes and cardiac fibroblasts of pressure-overloaded hearts. Upon overloading, no significant change in expression of miR-21 was observed in cardiomyocytes, while in fibroblasts, miR-21 was up-regulated. Increased levels of miR-21 target Spry1 resulting in increased activity of the MAPK/ERK signaling pathway, followed by fibroblast survival and growth factors release. In addition, fibroblast secretion of growth factors and increased fibrosis promote cardiomyocyte hypertrophy. [97].

Dilated cardiomyopathy belongs to the most common types of cardiac muscle disease in children leading to a significant number of pediatric heart transplantations. This disease is characterized by systolic dysfunction caused by the enlargement of the left ventricular chamber but normal wall thickness. Dilated cardiomyopathy accompanied by cardiomyocyte apoptosis leads to left ventricular remodeling and dysfunction [96,123,124]. One of the available biological models for the study of dilated cardiomyopathy is miR-1 double knockout mice. These modified animals are accompanied by the development of dilated cardiomyopathy and survive at the most to 17 postnatal days. In the heart of these animals was observed a decrease of miR-1 expression with the increase of fetal sarcomeric protein expression, which could change the stoichiometric composition of the sarcomere and decrease myocardial performance [96]. In addition, miR-1 targets the nuclear receptor gene estrogen-related receptor β, which is connected with fetal sarcomere-associated genes that commonly down-grade during adolescence [96,125].

### 5.2. Role of miR-1 and miR-21 in Diabetic Cardiomyopathy

Diabetic cardiomyopathy is defined as a chronic and irreversible cardiac complication in diabetic patients characterized by early diastolic dysfunction, cardiac hypertrophy, ventricular dilation, and systolic dysfunction, ultimately resulting in heart failure [126,127]. Among the miRs, miR-21 has often been found to be up-regulated, whereas miR-1 has been found to be mainly down-regulated under diabetic conditions in rodents [99,128]. Diabetes mellitus is accompanied by high glucose levels that could stimulate miR-1 expression in H9c2 cells through the MEK1/2 pathway and SRF, and the up-regulation of miR-1 suppresses Hsp60 expression, contributing to high glucose-mediated cardiomyocyte apoptosis [98]. Accordingly, miR-1 was assigned as a muscle-specific miR that impacts cardiomyocyte growth by negatively regulating calmodulin and nuclear factor in activated T cells (NFAT) signaling. In the diabetic heart, a target protein for miR-1 is the ryanodine receptor Ca^2+^ release channel complex (RyR2), which is located on the sarcoplasmic reticulum and is substantially important for Ca^2+^ transport and cardiomyocyte contraction. MiR-1 was found remarkably decreased in cardiomyocytes treated with high glucose, demonstrating that the down-regulation of miR-1 in diabetic heart is oxidative stress-dependent [99]. Silencing of miR-1 significantly inhibits apoptosis via the mitochondrial signaling pathway by regulating LXRα (liver X receptor, a key regulator of cholesterol homeostasis), the activation of which attenuates apoptosis in H9c2 cells. These results indicate that miR-1 represents an important player in the development of diabetic cardiomyopathy and provide novel insights into understanding the complex mechanisms involved in the pathogenesis of diabetic cardiomyopathy [129]. On the other hand, miR-21 levels were significantly augmented in high glucose-treated cardiac fibroblasts, which led to an increase in collagen synthesis and cardiac fibrosis through the JNK/SAPK and p38 signaling pathways by suppressing the expression of dual specific phosphatase 8 (DUSP8), which is involved in the proliferation and pro-collagen synthesis in cardiac fibroblasts. Moreover, inhibition of miR-21 reduced fibrosis via blocking the activation of the p38 signaling pathway, pointing to a crucial role of miR-21 in diabetic cardiomyopathy [100]. The analysis (GSE4745, microarray dataset) showed the down-regulation of Hk2 mRNA associated with high expression of its regulatory miRs (among other miR-21) in the left ventricles of rats exposed to a high glucose concentration, suggesting the important role of miR-21 in the pathophysiology of diabetic cardiomyopathy. Therefore, the up-regulation of rno-miR-21 and down-regulation of the Hk2 mRNA target could abolish cardioprotection and consequently lead to the development of cardiac complications, particularly those associated with hyperglycemia [130]. Additionally, miR-21 is also involved in neurohormone signaling in diabetic subjects, probably via the direct or indirect interaction with the 3′UTR of the atrial natriuretic peptide, which contributes to both cardiac hypertrophy and fibrosis resulting in diabetic cardiomyopathy [131]. Dai et al. [132] observed down-regulated heart tissue miR-21 in db/db mice, which contributed to diabetic cardiomyopathy. MiR-21 was also able to suppress gelsolin (GSN, an actin-binding protein that is a key regulator of actin filament), an important transcriptional cofactor in signal transduction in CVD. Moreover, gelsolin was referred to as a direct target of miR-21. Besides, exogenous miR-21 protected hearts against diastolic dysfunction. Thus attenuation of cardiac hypertrophy by decreased ROS production and improved NO release via gelsolin represent a new therapeutic strategy to treat diabetic cardiomyopathy [132].

### 5.3. Role of miR-1 and miR-21 in Viral Myocarditis

Viral myocarditis is defined as viral infection (mainly with the Coxsackie virus, adenovirus hepatitis virus, and HIV) of myocardial tissue leading to impaired heart function and heart failure [133,134,135]. MiR-21 has been shown to be altered in patients with acute myocarditis [133]. In the heart, during acute human and murine coxsackie B3 (CVB3) myocarditis, the up-regulation of miR-21 was detected [101]. For example, CVB3 infection decreased the expression of Cx43 by elevating the miR-1 level in mice with viral myocarditis [135]. In viral myocarditis was documented as highly expressed miR-21 in mice hearts. In vivo silencing of miR-21 was found to reduce inflammatory lesions and suppress T helper 17 cells (TH-17) differentiation in viral myocarditis in mice. Histological analysis of heart sections revealed that miR-21 inhibition using its inhibitors attenuated the severity of myocarditis. Expression of miR-21 transcripts correlated with IL-17 and RAR-related orphan receptor gamma (t) (RORγt) expression, suggesting that they may influence TH-17 transcription factor expression. Altogether, the data indicate that suppression of miR-21 could rescue hearts from CVB3 infection-caused myocarditis in mice [102].

**Figure 3:**
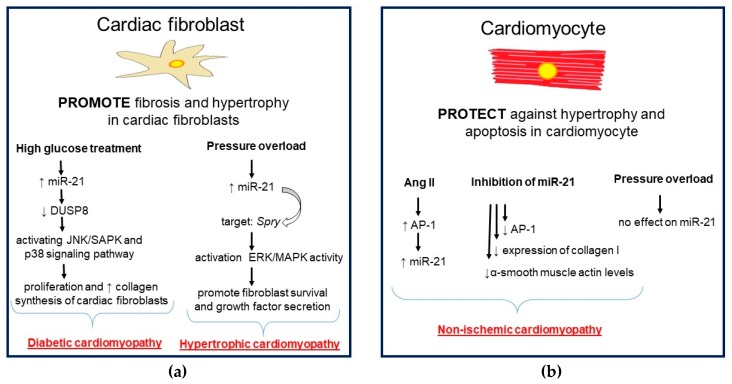
Cell-specific microRNA-21 (miR-21) expression in cardiomyopathies. In fibroblasts (**a**), miR-21 can promote cardiac fibrosis and cardiac hypertrophy. In cardiomyocytes (**b**), miR-21 can protect against hypertrophy and apoptosis. In fibroblasts of the pressure-overloaded heart (**a**), miR-21 is up-regulated, but not in cardiomyocytes (**b**). MiR-21 is involved in heart remodeling in non-ischemic and diabetic cardiomyopathy by promoting cardiac fibrosis. Figure assembled according to [95,97,100,122].

In conclusion, miR-1 and miR-21 have different roles in different types of cells and their expression has unique patterns in the dependence on types of cardiomyopathy (Figure 3). Moreover, as was mentioned in [10], miR-1-3p correlates with the left ventricular function of hypertrophic cardiomyopathy and can serve as a potential target for identifying the characteristics between hypertrophic and dilated cardiomyopathy. Findings documenting roles of miR-1 and miR-21 in different cardiomyopathies and other types of CVD are summarized in Table 1.

## 6. Role of miR-1 and miR-21 in Cardiotoxicity Induced by Cancer Treatment

Cancer is one of the leading causes of death worldwide and is usually treated by surgical intervention in combination with chemotherapy and/or radiotherapy. Despite the progress in the treatment methods, cancer treatment is also accompanied by several side effects, e.g., normal tissue toxicity such as cardiotoxicity. Several miRs including miR-1 and miR-21 have been shown to be implicated in cancer treatment-related cardiotoxicity [8,136].

### 6.1. Role of miR-1 and miR-21 in the Heart Injury Caused by Anthracyclines

Doxorubicin represents one of the most important anticancer drugs in the clinic. It is a member of the anthracyclines family. One of the principles of their antitumor action is the ability to inhibit topoisomerase II, which leads to double-stranded DNA breaks and thus hampers both cellular replication and transcription. Anthracyclines can directly intercalate into DNA, leading to the disruption of physiological protein/DNA interactions, and have been shown to produce ROS. As a result, exposed cells undergo DNA damage what potentially leads to cell death [137].

Unfortunately, the application of doxorubicin is connected to the disruption of cardiac activity, ranging from serious ventricular dysfunction to cardiomyopathies, and can develop into heart failure [138]. Cardiotoxicity develops either acutely or several years after anthracycline therapy. The incidence of acute cardiotoxicity is approximately 11%, while in the case of chronic doxorubicin cardiotoxicity, it is much lower at approximately 1.7%. The incidence of doxorubicin cardiomyopathy is primarily related to its dose. The incidence is about 4–5% when the cumulative dose of doxorubicin is 450–500 mg/m^2^, and 18% when the dose is 550–600 mg/m^2^ [139].

Many studies are aimed at investigating the mechanisms and identifying suitable biomarkers for early diagnosis of doxorubicin cardiotoxicity. Among them, miRs have been studied to be involved in doxorubicin-induced toxicity, including miR-1 and miR-21 [8]. Most of the studies have been performed on animal models. Desai et al. [111] studied the expression profiling of several miRs in a chronic doxorubicin cardiotoxicity mouse model. Cardiac injury was detected in mice exposed to an 18 mg/kg or higher cumulative doxorubicin dose, whereas investigation of hearts by light microscopy revealed cardiac lesions at 24 mg/kg of doxorubicin. In this study, the significant up-regulation of miR-21 in hearts correlated with a significant increase in plasma cTnT concentration at an 18 mg/kg cumulative dose or higher. Tong et al. [112] found that miR-21 protected cardiomyocytes against doxorubicin-induced apoptosis by targeting B-cell translocation gene 2 (*BTG2*), which belongs to the anti-proliferative gene family. Authors detected that the exposure of cardiac myocytes to doxorubicin at doses ranging from 0 to 4 µM for 24 h resulted in a significant dose-dependent increase in miR-21 expression level in H9C2 cells. Nishimura and his group [105] administered rats with a single intravenous dose of doxorubicin (30 mg/kg). MiR-1 showed up-regulation in rats’ plasma after doxorubicin treatment. However, plasma cardiac troponin-I and cTnT did not increase in doxorubicin rats, which points out to the fact that cardiac toxicity was not detected. The lack of histopathological findings in acute doxorubicin treatment is not surprising, as it would be expected in a chronic setting [8]. Razavi-Azarkhiavi et al. [108] explored the involvement of selected miRs in cardiotoxicity induced by doxorubicin. The results showed that doxorubicin down-regulated the expression of Bcl2 and PDCD4, and up-regulated Bax, caspase-3, and caspase-8 in mouse heart. Exposure of mice with 9 mg/kg of doxorubicin resulted in a significant increase in cardiac miR-1 expression. The expression level of miR-21 in mouse heart was also up-regulated due to doxorubicin (6 and 9 mg/kg). The study by Ruggeri et al. [107] concluded that the expression of specific plasma miRs reflects the presence of cardiac dysfunction in response to doxorubicin-induced injury. Among them, miR-1 was found down-regulated in comparison to controls treated with saline.

Several studies also focused on the investigation of the role of miRs in doxorubicin-induced cardiotoxicity in cancer patients. Rigaud et al. [106] evaluated circulating levels of miRs in breast cancer patients receiving doxorubicin treatment. They found that circulating levels of miR-1, as well as cTnI, increased during the treatment while overall left ventricular ejection fraction (LVEF) tended to decrease over 12 months. MiR-1 was associated with changes in LVEF. In another study, Leger and colleagues [109] investigated plasma miRs as potential biomarkers of cardiotoxicity in children and young adults treated with anthracycline chemotherapy. They screened 24 miRs in 24 anthracycline-based and nine non-cardiotoxic-agents-based chemotherapy cancer patients. MiR-1 was identified to be significantly up-regulated in anthracycline patients during experiment duration (24 h). MiR-1 expression correlated with the degree of anthracycline-induced injury as measured by cTnT. Breast cancer patients who developed abnormal LVEF after the completion of chemotherapy (doxorubicin) revealed the down-regulation of miR-1 in plasma [110].

In summary, most of the animal, as well as human, studies detected the up-regulation of miR-1 and miR-21 after doxorubicin treatment. Some studies have shown the opposite trend in miR-1 expression. The differences may be caused by different experimental conditions (dose, time, tissue vs. plasma).

### 6.2. Role of miR-1 and miR-21 in the Heart Injury Caused by Radiotherapy

Radiotherapy is another commonly used method for the treatment of oncological diseases. Radiation beams composed of gamma rays affect the cells by direct damaging of DNA, or indirectly through water hydrolysis. This indirect process leads to the formation of huge amounts of free radicals that are able to affect DNA and other important biological macromolecules in the cells. Both of these mechanisms of radiation action result in significant changes in the cells and may lead to its death. Unfortunately, despite a lot of very sophisticated techniques for the protection of tissues and cells surrounding tumorous cells, healthy uncancerous cells often receive some dose of radiation, leading to cardiomyopathies commonly called radiation-induced heart disease (RIHD) [140,141].

In the cardiovascular system, the main detrimental effect of radiation starts with the production of free radicals leading to the development of inflammatory processes [140]. Radiation exposure of the heart causes significant changes in the expression of many proteins like prostaglandins, prostacyclins, thromboxanes, and leukotrienes responsible for vasodilatation, vasoconstriction, and increasing microvascular permeability, as well as thrombosis [142]. It was demonstrated that irradiation also damages capillary endothelium, which results in the reduction of perfusion and ischemization of the myocardium [143,144]. Oncological patients treated by radiotherapy targeted into the mediastinum area (Hodgkin’s disease, breast cancer, or esophageal cancer) could receive an unwanted dose of radiation to the heart. These patients suffer from pericarditis, collagen, and fibrin deposition and the presence of exudate [145,146]. In the study by Barancik et al. [147], changes in the activation of MMP-2 in the irradiated rats were shown. Significant up-regulation of Cx43 is another change caused by irradiation [148] and cardiac hypertrophy as a compensatory mechanism that occurs [149].

Surova et al. [150] found up-regulated the overall expression of miRs in the cells by a higher expression of DROSHA and DICER enzymes. They revealed that these cells were more radioresistant compared to the cells with a lower expression of miRs. In the study by Kraemer et al. [151], global down-regulation of miRs by the inhibition of AGO2 and DICER enzymes was performed. The authors observed increased endothelial cell death after the exposure of cells with down-regulated miRs to gamma radiation. These data point to the conclusion that miRs have a significant impact on cellular response to irradiation.

Among the miRs connected with radiation-induced heart disease are also miR-1 and miR-21. Kura et al. [103] reported the down-regulation of miR-1 and the up-regulation of miR-21 in the rat myocardium six weeks after single irradiation of the mediastinum area with a total dose of 25 Gy. These results were confirmed in their next study in 2019 when RT-qPCR proved the down-regulation of miR-1 and the up-regulation of miR-21 in rats’ left ventricle after 10 Gy of irradiation in the mediastinal area. Slezak et al. [140] observed the up-regulation of miR-21 in rat hearts after irradiation of the mediastinum area with a single dose of 25 Gy. Similar results were measured by Viczenczova et al. [148] where the increased expression of miR-21 was associated with the up-regulation of total Cx43 protein expression in irradiated rat’s heart. The increased expression level of miR-21 was measured also in various oxidative stress-inducing conditions in human fibroblast cells [152] and this correlated with further findings [27,153,154].

To conclude, there are only a few studies dealing with the association of miR-1 and miR-21 with RIHD. In these, miR-1 was found down-regulated and miR-21 was up-regulated mainly due to the progression of oxidative stress-related damage. Radiotherapy and chemotherapy give cancer patients a significantly improved chance of survival. Unfortunately, these methods have a huge impact on the cardiovascular system. MiRs, as recently discovered potential key players in the regulation of cardiotoxicity, including miR-1 and miR-21, may have a role in managing undesired harmful effects of chemotherapy and radiotherapy on the heart. Findings documenting the roles of miR-1 and miR-21 in heart injury due to cancer treatment, as well as in other types of CVD, are summarized in Table 1.

## 7. Role of miR-1 and miR-21 in Cardioprotection

Extensive research has been performed to search for different cardioprotective interventions aimed to prevent or treat major types of CVD, mainly cardiac I/R injury associated with coronary artery disease and myocardial infarction, but also non-ischemic cardiac pathologies such as inflammatory, diabetic, and other cardiomyopathies, as well as cardiac injury due to other types of triggers such as anthracycline- or radiation-induced heart injury as a consequences of cancer treatments. Uncovering molecular mechanisms of particular CVD revealed molecular targets for potential cardioprotective interventions. Several ncRNAs, including miRs, were identified as potential molecular targets/mediators of cardioprotection induced by either endogenous or exogenous cardioprotective interventions, such as ischemic conditioning and pharmacological or non-pharmacological treatments. MiR-1 and miR-21 are two miRs detected to be involved in cardioprotection, mainly in I/R injury, but also in diabetic cardiomyopathy or viral myocarditis. The aim of this part of the paper is to briefly outline the potential roles of miR-1 and miR-21 in various cardioprotective approaches.

### 7.1. Role of miR-1 and miR-21 in Ischemic Conditioning

Ischemic conditioning is a powerful adaptive mechanism of the heart to I/R injury induced by cycles of brief ischemia of the coronary artery applied before (preconditioning) or after (postconditioning) the long-term ischemia. The conditioning stimuli can be applied also on the remote vessel or even remote organ to the heart called remote conditioning (pre-, per-, post-). Several miRs, including miR-1 and miR-21, have been proposed to be involved in ischemic conditioning either as mediators or targets for cardioprotection [155]. For instance, expression of both miR-1 and miR-21 were up-regulated in the heart tissue following ischemic preconditioning (IPC) in a mouse [156] as well as rat [157] Langendorff model of IPC. When the extracted and purified miRs from mouse IPC-hearts were injected in vivo into the left ventricular wall of intact mouse hearts, and 48 h later the hearts were subjected to a 30-min regional I/R by ligation of coronary artery followed by a 24-h reperfusion, the hearts were protected against I/R injury as manifested by the reduction of infarct size as compared with non-treated controls. This miR-induced protection was accompanied by up-regulated eNOS (endothelial nitric oxide synthase) and HSP70 (heat shock protein 70), suggesting that IPC-induced miRs (including miR-1 and miR-21) may trigger cardioprotection similar to the late IPC, possibly through eNOS and HSP70 [156]. However, a particular contribution of miR-1 and miR-21 to the effect of miR “cocktail” injected into hearts is impossible to detect from this study. MiR-21 was found to be up-regulated after IPC also in rats in vivo, and IPC-mediated cardiac protection against I/R injury was abolished by knockdown of cardiac miR-21 [83]. miR-21 also had a protective effect on hypoxia/reoxygenation-induced cell apoptosis in cultured cardiac myocytes, likely via inhibition of its target gene PDCD4 [83]. MiR-1, down-regulated due to I/R, has been shown up-regulated by ischemic postconditioning (IPostC) evoked by three cycles of 30/30s ischemia/reperfusion in an in vivo rat model of cardiac I/R. IPostC also up-regulated the expression of Bcl-2, down-regulated Bax, and caspase-9. The up-regulation of miR-1 (and miR-133a) in the study was also associated with decreased apoptosis of cardiomyocytes, altogether suggesting that miR-1 (and/or miR-133a) may play an important role in IPostC-induced cardioprotection by regulating apoptosis-related genes [158]. A very recent study in pigs revealed that IPostC induced by six cycles of 30/30s I/R immediately after a 90 min occlusion of coronary artery significantly down-regulated the plasma levels of miR-1 as compared to animals with IPC-AMI or AMI only [159]. IPostC in human patients undergoing double valve replacement also led to down-regulated miR-1 and up-regulated miR-21 in the right atrial muscle, and these changes were associated with up-regulated Bcl-2 mRNA and unchanged mRNAs for Bax and PDCD4, suggesting miR-1 and/or miR-21 to be involved in reducing apoptosis by IPostC in valve replacement surgery [160]. MiR-21 was found up-regulated in IPostC-induced cardioprotection in mice, while knockdown of miR-21 with antago-miR-21 abolished the protective effects of IPost. The protective effect of miR-21 in mouse IPostC cardioprotection was probably via activation of the PTEN/Akt pathway [161]. In remote ischemic preconditioning (RIPC), miR-1 was down-regulated in rat hearts following RIPC evoked by three cycles of 5/5 min occlusion/reperfusion of the femoral artery [157]. RIPC also prevented the up-regulation of miR-1 in the right atrium and preserved mitochondrial respiration during coronary bypass surgery in human patients [162]. In a study exploring time-dependent RIPC-affected expression of miR-1 following I/R in rats, miR-1 was down-regulated by RIPC maintained by four cycles of 5 min bilateral hind-limb ischemia without the following ischemia, as well as after I/R and RIPC followed by I/R after 2 h of reperfusion. After 6 h of reperfusion, RIPC led to the up-regulation of miR-1, but ischemia itself had no effect on miR-1 expression [163]. In cardioprotection by remote ischemic perconditioning (RIPerC) in patients with rheumatic valvular disease undergoing double valve replacement surgery, miR-1 was down-regulated and its putative target gene *Bcl-2* was up-regulated due to RIPerC, while miR-21 and its target gene PDCD4 were unchanged, suggesting miR-1 rather than miR-21 to be involved in mechanism of RIPerC-induced cardioprotection [164].

**Figure 4:**
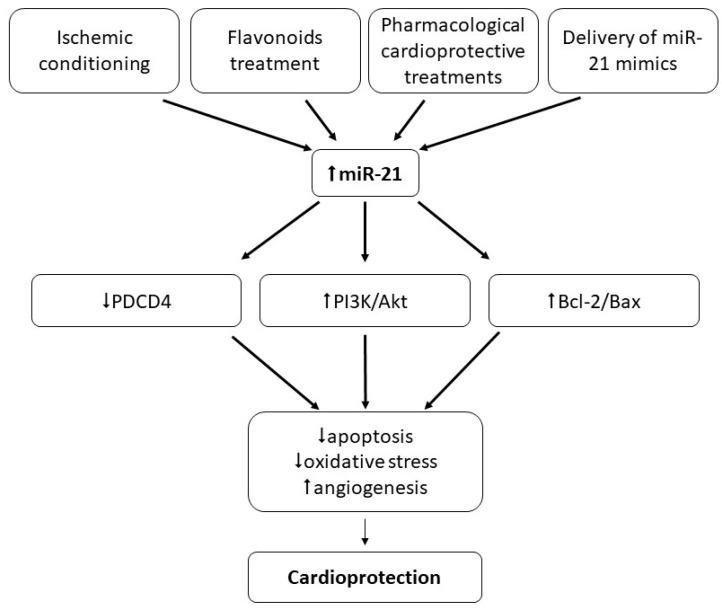
Role of miR-21 in cardioprotection. Up-regulation of miR-21 due to various interventions (e.g., ischemic conditioning, pharmacological and non-pharmacological, or miR-21 mimics) seems to serve cardioprotection mainly against I/R injury of the heart via inhibition of apoptosis and oxidative stress, and promoting angiogenesis.

In conclusion, miR-1 and miR-21 seem to be associated with different types of ischemic conditioning since their levels in both heart tissue and circulation were changed due to conditioning protocols; however, their exact roles in conditioning cardioprotection are not clear. It seems that while miR-21 exclusively plays a positive role in ischemic conditioning and contributes to cardioprotection (Figure 4), data documenting the role of miR-1 are controversial since this miR was found either up-regulated or down-regulated due to different conditioning protocols. Thus, further studies are needed to explore the biological significance of changes in miR-1 and miR-21 expression in ischemic conditioning-induced cardioprotection.

### 7.2. Role of miR-1 and miR-21 in Cardioprotection Other Than Ischemic Conditioning

In this part, we focus on the potential role of miR-1 and miR-21 in cardioprotective interventions other than ischemic conditioning, such as heat shock-induced cardioprotection, pharmacological cardioprotection, natural molecules-induced cardioprotection, as well as cardioprotection by delivery of miRs mimics.

It has been found that heat shock (HS) maintained by increasing the body temperature of mice to 42 °C for 15 min up-regulated the expression of several miRs including miR-1 and miR-21 in the tissue. When the miRs mixture isolated from HS hearts was intraperitoneally injected into the non-heat-shocked mice, it exerted cardioprotection against I/R manifested by a reduced infarct size. Moreover, chemically synthesized exogenous miR-21 also reduced infarct size, and miR-21-induced protection was abolished with miR-21 inhibitor co-treatment [165]. The potential role of miR-21 was suggested also in cardioprotection induced by anesthetic isoflurane. It was documented that isoflurane induced the up-regulation of miR-21 in both in vivo rat hearts and in vitro neonatal rat cardiomyocytes. The miR-21 increase was associated with the protection of cardiomyocytes against H_2_O_2_-induced oxidative stress-induced injury and the down-regulation of PDCD4 mRNA, suggesting the inhibition of PDCD4 to be involved in miR-21-induced cardioprotection [166]. Isoflurane protected via a miR-21-dependent mechanism mouse hearts exposed to I/R, potentially via the Akt/NOS/mPTP pathway [167]. The up-regulation of miR-21 expression was found also after chronic resveratrol treatment in rats, which was associated with cardioprotection against I/R [168]. The important role of miR-21 was suggested also in cardioprotection against cardiac I/R in vivo conferred by trimetazidine, a piperazine-derived metabolic agent. Trimetazidine exerted a protective effect against I/R injury accompanied by the up-regulation of miR-21, enhanced p-Akt, and improved Bcl-2/Bax ratio in rat hearts. Further, trimetazidine-induced cardioprotection against cardiac I/R injury was reversed by knockdown of miR-21 using anti-miR-21 plasmids. Thus, trimetazidine-induced cardioprotection against cardiac I/R might be mediated via miR-21, and PI3K/Akt and Bcl-2/Bax pathways [169]. Very recently, miR-21 was found to enhance the protective effect of loperamide, a drug used in diarrhea treatment, against hypoxia/reoxygenation injury in rat cardiomyocytes, associated with the reduction of ROS production and apoptosis, likely via regulating the A-kinase anchoring protein 8 (Akap8) and BRCA1-associated RING domain 1 (Bard1) expression [170]. MiR-21 up-regulation was found also in cardioprotection induced by thintra-myocardial adenovirus-mediated transplantation of extracellular superoxide dismutase (SOD) gene-modified bone marrow mesenchymal stromal cells into infarcted mouse hearts [171]. The nanoparticle delivery of miR-21 mimic to cardiac macrophages has been found to improve myocardial remodeling after MI demonstrated by enhanced angiogenesis and reduced hypertrophy, fibrosis, and apoptosis [75]. MiR-21 was documented to mediate also cardioprotection against diabetic cardiomyopathy-induced diastolic dysfunction, likely via targeting gelsolin, an actin-binding protein that is a key regulator of actin filament assembly and disassembly [132]. MiR-21 was found to play a protective role also against coxsackievirus B3 (CVB3) infection-induced heart infection, likely via targeting the MAP2K3/p-38 MAPK signaling pathway. In a mouse model of CVB3 infection, miR-21 pretreatment inactivated MAP2K3/p-38 MAPK signaling and exerted cardioprotection manifested by alleviated cell apoptosis and reduced necrosis in the heart, accompanied by reduced viral titers and the remarkably prolonged survival time of animals [172]. The overall picture of the cardioprotective action of miR-21 is outlined in Figure 4.

Regarding the role of miR-1 in cardioprotection, it was documented that daily administration of the traditional Chinese medicine tanshinone IIA for seven days led to cardioprotection associated with the down-regulation of miR-1, likely via inhibition of I/R-induced p-38 MAPK activation in an in vivo rat MI model [173]. The high glucose-induced down-regulation of Cx43 in neonatal cardiomyocytes was associated with the up-regulation of miR-1, and this was abolished with epigallocatechin-3-gallate (EGCG), thus suggesting that miR-1 suppression is involved in the cardioprotective effect conferred by EGCG [174]. Further, elevated expression of miR-1 due to H_2_O_2_-induced injury in H9c2 cells was down-regulated and cells were protected by insulin treatment. Since insulin abolished the detrimental effect of inhibition of the PI3K/Akt pathway, which further exacerbated miR-1-induced cell injury, it is suggested that PI3K/Akt is involved in the protective effect of insulin against miR-1-mediated injury under oxidative stress [175]. On the other hand, epirubicin-induced cardiotoxicity was associated with the down-regulation of miR-1, and phenolic compound paeonol up-regulated miR-1 expression and exerted the cardioprotective effect against epirubicin-induced heart injury manifested by improved cardiac dysfunction, abolished histopathological changes, alleviated inflammation, reduced apoptosis, and increased autophagy, likely via miR-1-mediated inhibition of PI3K/AKT/mTOR and nuclear factor kappa B (NF-κB) pathways activation [176]. Findings documenting roles of miR-1 and miR-21 in cardioprotection are summarized in Table 2.

## 8. Clinical implications of miR-1 and miR-21

Despite the large amount of recently published investigations showing associations between levels of ncRNAs and CVD, their potential as biomarkers or therapeutic targets is still a matter of debate. This appears to be true for the different types of ncRNAs, miRs not escaping the general rule. MiRs have been very largely investigated; a plethora of brilliant studies have been published, offering a detailed yet incomplete characterization of their role in CVD. The potential of circulating miRs to aid in the diagnosis of acute cardiac conditions such as myocardial infarction [177] or cardiac arrest [178] has been extensively reported. Yet, miRs are still not part of the toolbox of drugs, diagnostic, or prognostic markers of CVD available to clinicians. This is true for miRs in general, and for miR-1 and miR-21 in particular. The difficulty to translate research findings to clinical application has diverse and multiple explanations. For instance, novel miRs are not always identified from properly-sized cohorts of patients. Sample size is a critical determinant of biomarker studies, for which biostatisticians shall be enrolled from the study design until final data analysis. The reproducibility of findings is often hampered by the use of different experimental protocols that need to be homogenized. Blood sample type, storage, and processing are key to reproducible findings. MiR-1 and miR-21 are ubiquitously expressed, although miR-1 is enriched in muscle, and cardiac-specific miRs or myomiRs [177,179] may be more prone to reflect cardiac disease progression. However, other miRs not specific to the heart but strongly associated with inflammation have been reproducibly associated with cardiovascular events in the heart and the brain [180,181]. Interestingly, a recent study reported earlier rises in circulating levels of miR-1 after the transcoronary ablation of septal hypertrophy as compared to cardiac-enriched miRs, supporting its value as an adjunct marker of myocardial injury in predictive models, including established protein markers such as troponins [182]. It is known that women do not share the same level of risk for CVD as men, yet few sufficiently-powered studies have reported the capacity of miRs to be used as sex-specific biomarkers. All these limitations constitute future challenges that will have to be addressed before miRs can be added to the armada of clinically useful biomarkers of CVD. Ultimately, it is expected that better diagnostic and prognostic capacities will allow adapting healthcare strategies for the patient’s benefit. MiRs have the potential to aid in implementing personalized healthcare and reduce disease burden, in a sex-specific manner; however, the evidence is still missing.

## 9. Conclusions

The association between miR-1 and miR-21 with CVD has been extensively addressed, both from a functional angle and for their potential biomarker value. The majority of studies revealed that while miR-1 plays a potentially detrimental role in cardiac I/R injury, miR-21 seems to be cardioprotective in I/R-challenged hearts. On the other hand, diverse changes in miR-1 and miR-21 have been found in other types of heart injury like anthracycline or radiation-induced cardiotoxicity or different cardiomyopathies. Moreover, changes in these two miRs due to CVD may differ depending on the cell type within the heart. Thus, a better characterization of the role of these two miRs in CVD development may open new avenues to design next-generation therapeutic and predictive strategies. Whether miR-1 and miR-21 will soon be translated to clinical application remains uncertain, but the hope is there.

## Figures and Tables

**Table 1:** Role of miR-1 and miR-21 in different types of CVD.

Type of CVD	miR	Findings	Reference
Ischemia-reperfusion (I/R) injury	miR-1	↓miR-1 in heart tissue in response to I/R in rats, mice, and infarcted human hearts	[48,49,50,52,53]
		↓miR-1 in H9c2 cells and neonatal cardiac myocytes in response to H/R	[48,51]
		↑miR-1 in remote myocardium compared to infarcted zone or healthy hearts in infarcted human hearts	[54]
		↑levels of circulating miR-1 after AMI in pigs and humans	[58,59]
		miR-1 overexpression exacerbated cardiac I/R injury in transgenic mice	[55]
		miR-1 inhibition protects against I/R (H/R) injury in rats, mice, and H9c2 cells	[48,55,56]
	miR-21	↓miR-21 in in infarct areas, ↑miR-21 in borderline areas in I/R model in rats	[62]
		↑miR-21 in infarct zone of mouse hearts on days two and seven post-MI	[63]
		↓miR-21 in heart tissue in the first and second week but unchanged in the fourth week post-AMI in mice	[64]
		↑miR-21 in mouse neonatal cardiomyocytes, H9c2, HL-1, and HCM cells exposed to H/R	[65]
		diverse time-dependent changes in circulating miR-21 in post-MI patients	[76]
		↑circulating miR-21 in patients with CAD undergoing dobutamine stress echocardiography	[82]
		↑miR-21 in serum of elderly patients with AMI correlated with levels of CK-MB and cTnI	[68]
		↓miR-21 in rat neonatal cardiomyocytes exposed to OGD and ↑PDCD4, ↑apoptosis, ↑ROS	[66]
		miR-21 protected cultured cardiac myocytes against H/R-induced apoptosis via ↓PDCD4	[83]
		↑miR-21 in the infarct zone promoted myocardial fibrosis post-MI in mice	[71]
		↑miR-21 in heart atria was associated with ↑atrial collagen content in patients with AF	[72]
Cardiac arrhythmias	miR-1	↑miR-1 expression in the heart associated with ↑arrhythmogenesis in rodents, dogs, and humans	[84,85,86,87]
		↓miR-1 expression in heart tissue in patients with age-associated AF and in patients with permanent AF undergoing heart surgery	[88,89]
	miR-21	↑miR-21 in heart tissue in patients with AF	[90]
		↓plasma levels of miR-1 in patients with AF	[91]
		↑miR-21 promote fibrosis in AF in rodents	[92,93,94]
Non-ischemic cardiomyopathy	miR-21	↑miR-21 expression in mice heart with transverse aortic constriction, ↓PDCD4	[95]
		↑miR-21 expression in NRCM with Ang II	[95]
Dilated cardiomyopathy	miR-1	unchanged miR-1-3p expression in left ventricles in humans with dilated cardiomyopathy	[10]
		↓miR-1 in heart of (miR-1 dKO) mice	[96]
	miR-21	↑miR-21 in left ventricles in patients with dilated cardiomyopathy	[10]
Hypertrophic cardiomyopathy	miR-1	↓miR-1-3p in human left ventricles	[10]
	miR-21	unchanged miR-21 in left ventricles in patients with hypertrophic cardiomyopathy	[10]
		↑miR-21 in fibroblasts of the pressure-overloaded heart	[97]
		unchanged miR-21 in cardiomyocytes of the pressure-overloaded heart	[97]
Diabetic cardiomyopathy	miR-1	↑miR-1 regulation in H9C2 in high glucose	[98]
		↓miR-1 in cardiomyocytes treated with high glucose	[99]
	miR-21	↑miR-21 in high glucose-treated cardiac fibroblasts	[100]
Viral myocarditis	miR-21	↑miR-21 in human and murine coxsakcie B3 myocarditis	[101,102]
Radiation-induced heart disease	miR-1	↓miR-1 in left ventricle six weeks after 25 Gy and 10 Gy irradiation of mediastinum area	[103,104]
	miR-21	↑miR-21 in left ventricle six weeks after 25 Gy and 10 Gy irradiation of mediastinum area	[103,104]
Anthracyclines-induced cardiomyopathy	miR-1	↑miR-1 in blood plasma of rats after doxorubicin treatment	[105]
		↑miR-1 in blood plasma of cancer patients after doxorubicin treatment	[106]
		↓miR-1 in blood plasma of rats after doxorubicin-induced injury	[107]
		↑miR-1 in heart tissue of rats after doxorubicin treatment	[108]
		↓miR-1 in blood plasma of cancer child and young adult patients after anthracycline treatment	[109]
		↓miR-1 in blood plasma of breast cancer patients after doxorubicin treatment	[110]
	miR-21	↑miR-21 in mice hearts after doxorubicin treatment	[108,111,112]

Abbreviations: CVD: cardiovascular disease; I/R: ischemia/reperfusion; H/R: hypoxia/reoxygenation; AMI/MI: (acute) myocardial infarction; STEMI: ST elevation myocardial infarction; PCI: percutaneous coronary intervention; CM: cardiac myocytes; HCM: human cardiomyocyte cell line; PDCD4: programmed cell death protein 4; AP-1: activator protein 1 (downstream molecule of PDCD4); ROS: reactive oxygen species; IS: infarct size; LV: left ventricle; CPC: cardiac progenitor cell; AF: atrial fibrillation; CAD: coronary artery disease; TASH: transcoronary ablation of septal hypertrophy; OGD: oxygen-glucose deprivation (simulated ischemia).

**Table 2:** Role of miR-1 and miR-21 in cardioprotection.

Type of Intervention	miR	Findings	Reference
Ischemic conditioning	miR-1	↑miR-1 in heart tissue after IPostC in rats	[158]
		↓miR-1 in plasma after IPostC in pigs	[159]
		↓miR-1 in right atria after IPostC in human patients undergoing cardiac surgery	[160]
		↓miR-1 in heart tissue after RIPC in rats	[157]
		↓miR-1 by RIPC in in vivo cardiac I/R model in rats	[163]
		RIPC prevented up-regulation of miR-1 in right atria and preserved mitochondrial respiration during heart surgery in humans	[162]
		↓miR-1 by RIPerC during heart surgery in humans	[164]
	miR-21	↑miR-21 in heart tissue after IPC and IPostC in animal models and humans	[83,160,161]
		knockdown of miR-21 abolished cardioprotective effects of IPost in mice	[161]
		knockdown of cardiac miR-21 abolished IPC-mediated cardioprotection against I/R in rats	[83]
Pharmacological	miR-1	insulin protected against miR-1-mediated H_2_O_2_-induced injury in H9c2 cells	[175]
	miR-21	isoflurane-induced up-regulation of miR-21 associated with ↓PDCD4 protected cardiomyocytes against H_2_O_2_ injury	[166]
		isoflurane protected mouse hearts exposed to I/R via miR-21 and Akt/NOS/mPTP	[167]
		trimetazidine-induced ↑miR-21 accompanied by cardioprotection against I/R, ↑p-Akt and ↑Bcl-2/Bax in rats. Cardioprotection reversed by anti-miR-21	[169]
		miR-21 enhanced protective effect of loperamide against H/R injury in rat cardiomyocytes associated with ↓ROS and ↓apoptosis	[170]
Non-pharmacological	miR-1	down-regulation of miR-1 by traditional Chinese medicine Tanshinone IIA led to cardioprotection via inhibition of I/R-induced p-38 MAPK in rats	[173]
		phenolic compound paeonol exerts cardioprotection against epirubicin-induced heart injury via regulation of miR-1, PI3K/AKT/mTOR and NF-κB	[176]
	miR-21	resveratrol-induced up-regulation of miR-21 associated with protection against I/R in rats	[168]
miR transfection and delivery	miR-21	adenovirus miR-21 transfection decreased IS via targeting PDCD4/AP-1	[62]
		adenovirus miR-21 transfection improved LV remodeling & ↓apoptosis in cardiac I/R in rats	[67]
		lentivirus miR-21 transfection induced cardioprotection against I/R in mice manifested by ↓IS, ↓fibrosis and ↓apoptosis	[64]
		miR-21 transfection to human cardiomyocytes ↓apoptosis via JNK/p38-MAPK/caspase-3	[68]
		chemically synthesized exogenous miR-21 reduced IS in mice, miR-21-induced protection was abolished with miR-21 inhibitor co-treatment	[165]
		nanoparticle delivery of miR-21 to cardiac macrophages post-MI promoted angiogenesis, reduced hypertrophy, fibrosis, and apoptosis in the remote myocardium	[75]
		miR-21 pretreatment exerted cardioprotection against CVB3 infection via targeting MAP2K3/p38-MAPK in mice	[172]
Exosomal miR	miR-21	depletion of exosomal miR-21 reduced protective effect of conditioned medium in H_2_O_2_-induced oxidative stress in cardiomyocytes, and in rat hearts exposed to AMI	[69]
		↑miR-21 in CPC-derived exosomes prevented apoptosis in H9c2 cells via ↓PDCD4	[70]

Abbreviations: I/R: ischemia/reperfusion; H/R: hypoxia/reoxygenation; AMI/MI: (acute) myocardial infarction; PDCD4: programmed cell death protein 4; AP-1: activator protein 1 (downstream molecule of PDCD4); ROS: reactive oxygen species; IS: infarct size; LV: left ventricle; CPC: cardiac progenitor cell; AF: atrial fibrillation; IPC: ischemic preconditioning; IPostC: ischemic postconditioning; RIPC: remote ischemic preconditioning; RIPerC: remote ischemic preconditioning; CVB3: coxsackievirus B3.
